# Over-Mutated Mitochondrial, Lysosomal and TFEB-Regulated Genes in Parkinson’s Disease

**DOI:** 10.3390/jcm11061749

**Published:** 2022-03-21

**Authors:** Eulàlia Segur-Bailach, Olatz Ugarteburu, Frederic Tort, Laura Texido, Celia Painous, Yaroslau Compta, Maria José Martí, Antonia Ribes, Laura Gort

**Affiliations:** 1 Secció d’Errors Congènits del Metabolisme-IBC, Servei de Bioquímica i Genètica Molecular, CDB, Hospital Clínic de Barcelona, IDIBAPS, CIBERER, 08028 Barcelona, Spain; laiasegur@gmail.com (E.S.-B.); ougarteburu@gmail.com (O.U.); ftort@ciberer.es (F.T.); lauratexido79@gmail.com (L.T.); 2 Parkinson’s Disease & Movement Disorders Unit, Hospital Clínic de Barcelona, IDIBAPS, CIBERNED, European Reference Network for Rare Neurological Diseases (ERN-RND), Institut de Neurociències, Universitat de Barcelona, 08036 Barcelona, Spain; painous@clinic.cat (C.P.); ycompta@clinic.cat (Y.C.); mjmarti@clinic.cat (M.J.M.)

**Keywords:** Parkinson’s disease, lysosomal, mitochondrial, TFEB, risk-factor, WES, lysosomal diseases, mitochondrial function

## Abstract

The association between Parkinson’s disease (PD) and mutations in genes involved in lysosomal and mitochondrial function has been previously reported. However, little is known about the involvement of other genes or cellular mechanisms. We aim to identify novel genetic associations to better understand the pathogenesis of PD. We performed WES in a cohort of 32 PD patients and 30 age-matched controls. We searched for rare variants in 1667 genes: PD-associated, related to lysosomal function and mitochondrial function and TFEB-regulated. When comparing the PD patient cohort with that of age matched controls, a statistically significant burden of rare variants in the previous group of genes were identified. In addition, the Z-score calculation, using the European population database (GnomAD), showed an over-representation of particular variants in 36 genes. Interestingly, 11 of these genes are implicated in mitochondrial function and 18 are TFEB-regulated genes. Our results suggest, for the first time, an involvement of TFEB-regulated genes in the genetic susceptibility to PD. This is remarkable as TFEB factor has been reported to be sequestered inside Lewy bodies, pointing to a role of TFEB in the pathogenesis of PD. Our data also reinforce the involvement of lysosomal and mitochondrial mechanisms in PD.

## 1. Introduction

Parkinson’s disease (PD) is the second most prevalent neurodegenerative disorder, characterized by the accumulation of alpha-synuclein (ASYN), which plays an important role in the degeneration of dopaminergic neurons [1]. Monogenic forms of PD can explain 30% of familial PD and approximately 3–5% of sporadic cases. However, the majority of PD cases are considered to have a polygenic origin. In addition, PD can be caused by both genetic and environmental factors, showing an age-dependent prevalence form. In recent years, genome-wide association studies have compiled a list of PD-associated genes, most of them encoding for proteins involved in lysosomal function, autophagy pathways, endocytosis, mitochondrial physiology and immune response [2,3,4].

The association between lysosomal storage diseases (LSDs) and PD has been widely described in several studies. In fact, both groups of diseases are characterized by the accumulation of non-degraded substrates which triggers the pathologic cascade and defects on lysosomal proteins that may alter the ASYN clearance [5]. Several studies highlight the elevated frequency of GBA mutations in PD patients, accounting for 2–30% of PD cases. Heterozygous carriers of GBA mutations may have a five-fold increased chance of developing PD compared to the general population, while Gaucher disease patients have an increased risk of 10 to 20 times of developing PD [6]. In the past few years, large sequencing studies have put into consideration the higher risk of PD among carriers of mutations in genes involved in LSDs, showing at least one putative pathogenic variant in 56% of PD cases [5,7]. Furthermore, rare genetic variants in lysosomal genes, such as ATP13A2, LAMP1, TMEM175 and VPS13C have been reported to be over-represented in PD patient cohorts [8].

Mitochondrial dysfunction has also been associated with neurodegeneration and to PD pathogenesis. Koziorowski et al., suggested that both mitochondrial dysfunction and ASYN deposits may be co-dependent phenomena [9]. In particular, mitochondrial ASYN accumulation results in decreased complex I activity and promotes mitophagy. Consequently, reduced ATP synthesis and increased oxidative stress has been observed in PD patients. This mitochondrial alteration triggers the degeneration of PD neurons in mouse models [10]. Indeed, disrupted mitochondrial function, particularly complex I and IV defects, together with oxidative damage, have been identified in post-mortem studies of PD patients and animal models [11].

Interestingly, mitochondrial toxins and pesticides such as 1-methyl-4-phenyl-1,2,3,6-tetrahydropyridine (MPTP), which leads to the inhibition of the mitochondrial respiratory chain, have been reported to cause parkinsonism in humans as well as in animal models [12]. Moreover, alterations in POLG, a gene involved in mtDNA maintenance, has been found to influence the risk of idiopathic PD, whereas mutations in other genes encoding mitochondrial proteins, including SNCA, LRRK2, PRKN and PINK1, have been associated with hereditary forms of PD [13,14].

The role of lysosomal and mitochondrial variants in PD pathogenesis is not completely understood. Nevertheless, the main hypothesis suggests that alterations in these genes could cause a failure of endolysosomal and autophagic pathways [6,15]. In fact, autophagy is the main degradation pathway of ASYN, and the presence of aggregates has been associated with an accumulation of autophagosomes and a reduction of lysosomal markers, suggesting a defect in the autophagy-lysosome pathways [16,17,18]. Related to that, the transcription factor EB (TFEB), a master regulator of lysosomal biogenesis and autophagy, has been reported to be sequestered by ASYN inside Lewy Bodies. Interestingly, the cytoplasmic retention of TFEB would block the expression of lysosomal and autophagic genes, inhibiting the ASYN clearance [16].

The aim of this study was to identify new genetic risk factors associated with PD pathogenesis. In particular we focused on the potential role of mutations in genes involved in lysosomal function, autophagy, mitochondrial physiology and TFEB-regulated genes.

## 2. Materials and Methods

### 2.1. Patients and Controls

We analysed a cohort of 32 unrelated patients (18 males and 14 females) with PD diagnosed at the Neurology Department of Hospital Clínic, Barcelona, according to the Movement Disorder Society (MDS) diagnostic criteria for Parkinson’s disease [19]. They were aged between 41 and 86, but they were diagnosed between 33 and 82. Genetic studies were not previously performed in any of these patients. In this study, 30 age-matched healthy unrelated volunteers were used as controls. The clinical presentation and the genetic findings of the PD patients are described in Supplementary Table S1. The genetic findings in the healthy controls are described in Supplementary Table S2.

### 2.2. Whole-Exome Sequencing

Genomic DNA from patients and control individuals was isolated from blood samples following the manufacturer’s recommendations (QiAmp DNA Mini Kit, QIAGEN, Hilden, Germany). Whole-exome sequencing (WES) was performed at the National Centre for Genomic Analysis (CNAG-CRG, Barcelona, Spain). Exome enrichment was performed using the SeqCap_EZ_Exome_v3 kit (Nimblegen), followed by sequencing using the Illumina HiSeq 2000 genome analyser platform. Sequencing and analysis of the primary data (FASTQ files) were performed at CNAG-CRG using a self-developed pipeline.

### 2.3. Genetic Data Analyses

The VCF files were annotated and analysed using the BaseSpace Variant Interpreter platform (Illumina©, San Diego, CA, USA). Genetic variants were filtered according to the following criteria: 

In all the samples, we searched for the presence of rare variants (minor allele frequency, MAF <4%) in 1667 genes belonging to the following virtual gene panels: (a) 53 genes associated with PD, including 11 genes associated with PD monogenic forms, (b) 138 genes related to lysosomal function, including 56 genes associated with LSD, (c) 428 TFEB-regulated genes, 63 of them related to autophagy, and (d) 1158 genes related to mitochondrial function annotated into Mitocarta 2.0 [20,21]. The TFEB-regulated genes were selected according to the bibliography [22,23] (Supplementary Table S3). Due to overlapping among analysed groups, some of the genes were included in more than one panel.

**Figure 1:**
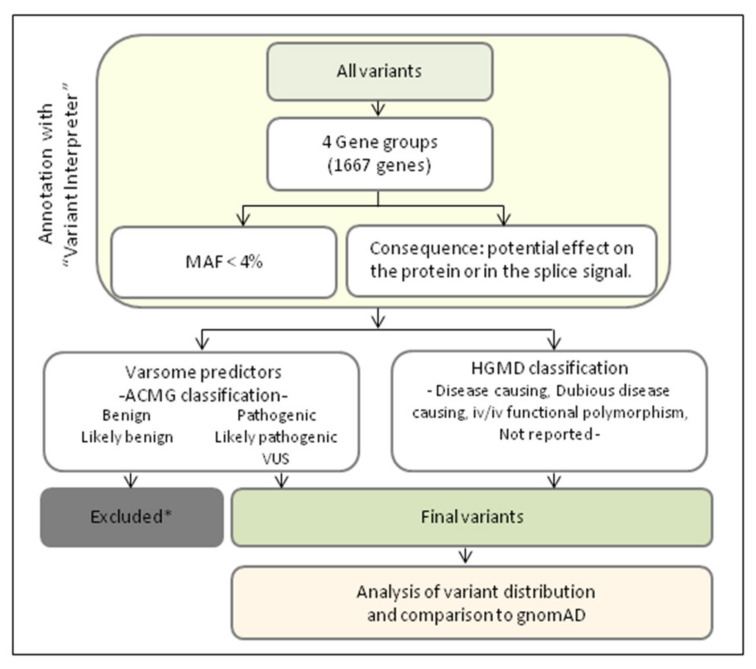
Flowchart of the analytical strategy used in this study. * Except those variants classified in the HGMD as disease causing, dubious disease causing or iv/iv functional polymorphism.

The flowchart of the whole strategy used in this study is shown in Figure 1. The variants were further filtered according to their functional consequences; prioritization was established in those with a potential effect on the protein or those, causing splicing defects. The variants were classified according to the American College of Medical Genetics (ACMG) recommendations [24]. Using the Varsome platform [25,26]. The identified variants were also classified according to the Human Gene Mutation Database^®^ Professional 2020.3 (HGMD) [27]. The variants not annotated in HGMD were classified as non-reported.

We included in the analysis those rare variants already reported or those predicted to cause some effect on the protein. The inclusion criteria were as follows: according to ACMG, we included variants classified as pathogenic, likely pathogenic or Variant of Uncertain Significance (VUS). VUS are variants with insufficient evidence to determine if they are disease causing or not; but some of them turn out to be pathogenic when functional studies are performed; for that reason, VUS have been included. According to HGMD, we included variants classified as disease causing, dubious disease causing or in vitro or in vivo functional polymorphisms. This last classification is for those polymorphisms reported to affect the structure, function or expression of the gene (or gene product), but with no disease association reported so far.

### 2.4. Statistical Analysis

When comparing the number of variants identified in our PD cohort versus the control cohort, we performed a student’s *t*-test using a parametric method assuming that our data follow a normal distribution. Values of *p* < 0.05 were considered statistically significant. To elucidate if the variants of our PD cohort were significantly overrepresented, we used the Z-score statistic test using the frequency reported in the European population database (GnomAD, [28]). For this analysis, we selected variants that were annotated in HGMD or those variants classified as “pathogenic” or “likely pathogenic” according to the ACMG criteria. Frequency variants with Z-score > 3 were considered significant.

## 3. Results

**Figure 2:**
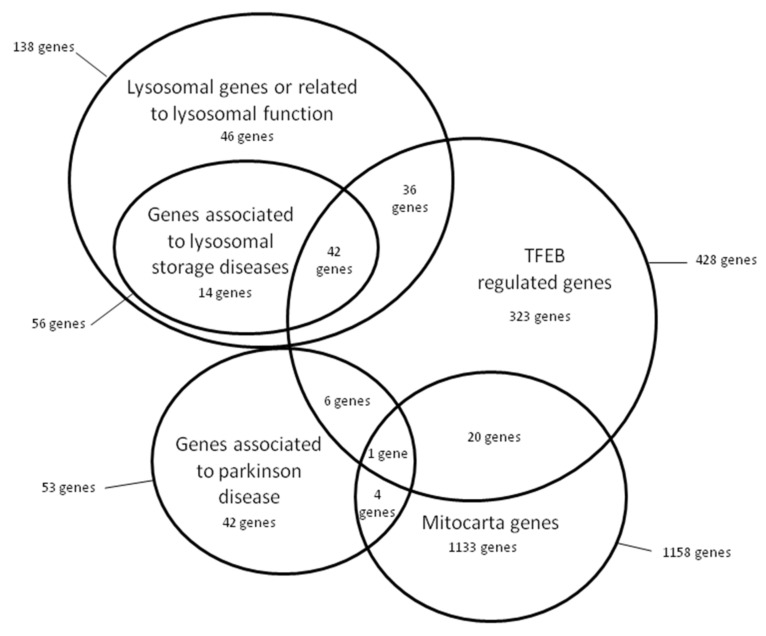
Graphical representation by groups of the 1667 genes analysed in this study and the intersection among them.

We performed a comprehensive genetic study in a cohort of 32 PD patients and 30 age-matched healthy controls. We first searched for mutations in genes previously reported to be causative or associated with PD. We then searched for rare variants in lysosomal, mitochondrial function and TFEB-regulated genes. In total, we screened 1667 genes (Figure 2, Supplementary Table S3).

### 3.1. Identification of Mutations in PD-Associated Genes

This study was performed in a subset of 11 genes responsible for the monogenic forms of PD, and 42 genes previously reported to be associated with PD (Supplementary Table S3). Among the first group of genes, we identified previously reported pathogenic mutations in LRRK2 and PRKN in two patients (Table 1).

Consequently, the genetic cause of the disease is now established in these two patients. Concerning the second group of genes, variants in a total of nine patients were identified in six genes. All of the variants but three were classified as VUS. Therefore, functional studies are necessary to fully demonstrate the disease causality of these variants. The other three variants, previously reported as disease-causing variants, were found in SPR [29] and POLG [30], respectively (Table 1). Interestingly, the SPR gene is localized in the autosomal dominant Parkinson’s disease-3 (PARK3) locus, and there is accumulated evidence suggesting its association to early-onset PD. In fact, this patient was 53 years old when the first symptoms appeared. Therefore, it might be that SPR mutation could influence his presentation. On the contrary, mutations identified in POLG in one of the patients, although reported as pathogenic, segregation studies demonstrated that the healthy mother harbours the same two mutations and the father does not carry any mutation. Therefore, both mutations segregate in the same allele.

### 3.2. Over-Mutation in Lysosomal, Mitochondrial and TFEB-Regulated Genes in PD 

We next investigated the number of rare variants in these three groups of genes. Results of the genes associated to lysosomal function or to LSD (138 genes) showed that 45% of the PD patients carried four or more variants, while this finding was only present in 17% of the controls (Supplementary Figure S1B). Interestingly, if we only take into account the 56 genes reported to be associated with LSD, the percentage increases to 64% in PD versus 40% in the control cohort (Supplementary Figure S1A). The analysis of mitochondrial genes included in the Mitocarta (1158 genes) revealed that 76% of PD patients carried five or more variants versus 39% of the control cohort (Supplementary Figure S1C). Interestingly, the analysis of the 428 TFEB-regulated genes, showed that 85% of the PD patients harbour six or more variants, versus 45% of the control cohort (Supplementary Figure S1D). We also investigated the number of variants in the different groups of genes, taking into account the age of onset by comparing patients diagnosed before and after the age of 50 years. We did not find significant differences (Supplementary Figure S2A).

**Figure 3:**
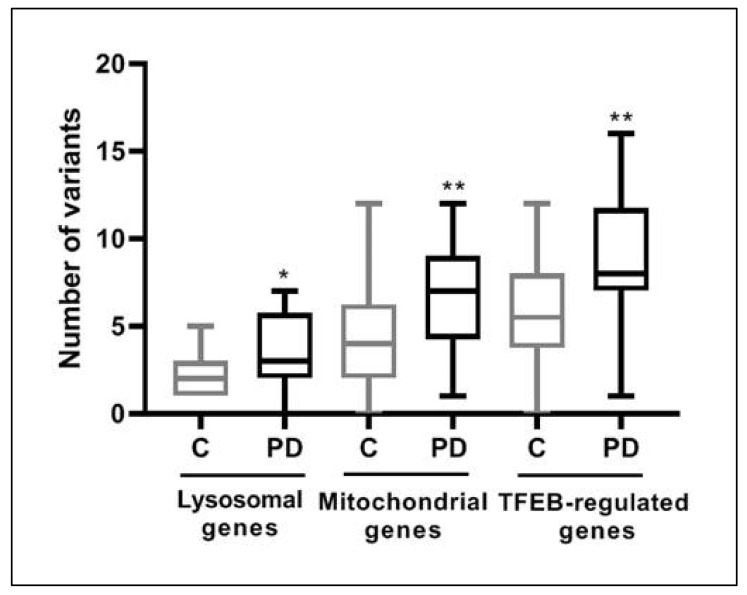
Number of variants carried by PD patients and control individuals for each group of genes. PD, Parkinson’s disease patient cohort; C, control individuals cohort. * *p* < 0.05, ** *p* < 0.01.

On the bases of previous observations, we decided to compare the mean of the number of variants carried by the cohort of PD patients with the cohort of age-matched controls for the three selected group of genes. Results showed significant differences in all the selected groups; *p* values for these groups were: lysosomal (*p* < 0.05), mitochondrial (*p* < 0.01) and TFEB-regulated genes (*p* < 0.01) (Figure 3). To calibrate potential artifacts in the analyses, we also compared the number of synonymous variants (presumably likely benign or benign) present in PD patients and controls in the three group of genes. The differences between them were not statistically significant (Supplementary Figure S2B).

We also analysed the frequency of the variants identified in the cohort of PD patients and compared it to the frequency reported in the database of European population (GnomAD). Using the Z-score statistic test, we identified an over-representation of 53 variants in 36 of the analysed genes (Table 2, Supplementary Table S1). Interestingly, 18 of them were TFEB-regulated genes, while the other 18 were non-regulated by TFEB, of which 11 were of mitochondrial origin, 6 were associated with PD and only one was of lysosomal origin (Table 2).

## 4. Discussion

Parkinson’s disease is one of the most common neurodegenerative disorders in humans. The genetic bases of PD are complex, and only a small percentage of cases have a monogenic cause. The majority of cases are assumed to have a polygenic origin, and in fact, alterations in a plethora of genes have been associated with increased susceptibility to the disorder [1].

The present study allowed us to identify the genetic cause of the disease in two patients in which we identified reported mutations in PRKN and LRRK2. These two genes are associated with monogenic PD. We also identified the potential cause of the disease in nine additional individuals harbouring variants in other PD-associated genes (Table 1). These results highlight the usefulness of WES to identify the disease-causing genetic defect in some patients, allowing them access to genetic counselling. Interestingly, we identified one patient with a reported complex allele carrying two mutations in cis c.[752C>T; 1760C>T] (p.[Thr251Ile; Pro587Leu]) in POLG encoding a protein involved in mtDNA maintenance [31]. Biallelic mutations in this gene have been associated with mitochondrial DNA depletion syndrome and also to early-onset PD [32,33]. However, as both identified mutations segregate in the same allele, it is not likely to be the cause of the disease in our patient. However, the clinical consequences of this complex allele need to be further clarified because heterozygous POLG mutations have been associated with an autosomal dominant form of progressive external ophthalmoplegia (adPEO) [34]. In this case, the identification of one mutated allele is enough to trigger the pathology. Individuals with adPEO presented in the adulthood and the main symptoms included myopathy, sensorineural hearing loss, hypogonadism, cataracts, axonal neuropathy, ataxia and, interestingly, parkinsonism [35,36,37]. In addition, the complex allele c.[752C>T; 1760C>T] identified in our patient has been previously associated with neurosensorial hipoacusia, fatigue, heart block and intellectual disability [30]. The fact that our patient presented an early onset form of PD provides evidence about the complexity and diversity of genotype-phenotype correlations associated with POLG mutations.

It is also interesting to highlight that two out of the three heterozygous carriers of PRKN mutations and three out of the six carriers of ATP7B variants had an early onset presentation of the disease, being diagnosed before 50 years of age (Supplementary Table S1). These data are in agreement with previous observations [38,39,40].

Recent studies have demonstrated an excessive burden of mutations in genes associated with lysosomal function in idiopathic PD patients [7]. Here we expanded the genetic analysis to 138 genes associated with lysosomal function, including those associated with lysosomal storage disease (LSD). Despite the number of analysed genes being higher than other published studies, the median number of variants detected in lysosomal genes remains similar to previous reports [7,8]. Moreover, particular genetic variants identified in 11 lysosomal genes have been found to be overrepresented when compared to the European population. As a matter of fact, some of them, such as genetic variants in *GBA*, *SMPD1* and *ATP13A2*, have been broadly associated with PD [2]. Therefore, our results corroborate the idea that lysosomal genes are over-mutated in PD patients.

At present, several lines of evidence suggest that loss of function of lysosomal proteins may also lead to autophagy deregulation, linking this process to the development of PD [41]. Interestingly, an important number of genes associated with autophagic processes are regulated by TFEB, a transcription factor that acts as a master regulator of lysosomal biogenesis and autophagy, among others. It has been shown that TFEB is sequestered by ASYN inside Lewy bodies in PD patients. The cytoplasmic retention of TFEB prevents its nuclear translocation and blocks the expression of lysosomal and autophagic genes, inhibiting the ASYN clearance [16]. In addition, we hypothesized that if PD patients had variants in the genes regulated by TFEB, perhaps their function could be altered and become an additional susceptibility factor for the development of PD. The potential role of these genes as a genetic risk factor for PD has not yet been explored. Therefore, we thought it could be of interest to include the analysis of TFEB-regulated genes in our study. Interestingly, we found a significant increase of variants in TFEB-regulated genes. Moreover, particular variants in 18 of these genes were overrepresented. As indicated in Table 2, half of the identified genes showing overrepresented variants are TFEB-transcriptional targets, including 10 out of the 11 lysosomal genes. Remarkably, we found a significant overrepresentation of the *SQSTM1* c.1175C>T variant (p.Pro392Leu). This gene encodes p62, a protein implicated in the degradation of missfolded proteins or aggregates, and its dysfunction causes an increase of ASYN as well as other protein aggregates [18]. The fact that p62 is also involved in the PINK1-PRKN pathway and in the regulation of LRRK2 stability suggests that p62 deficiency might contribute to the pathogenesis of PD [42,43]. Therefore, we speculate that PD patients carrying mutations in SQSTM1 would have an impaired basal autophagy, and perhaps might be considered as a risk factor for the development of PD. However, functional data are necessary to elucidate the potential role of SQSTM1 mutations in PD susceptibility.

We have also analysed our PD cohort for the presence of variants in the Mitocarta-annotated genes. Similar to that seen for lysosomal and TFEB-regulated genes, we observed an increased number of variants in mitochondrial-related genes. Interestingly, most of the mitochondrial genes carrying overrepresented variants encode proteins involved in mtDNA maintenance and in the OXPHOS system, particularly complexes I and IV (Table 2). These findings are in agreement with the previously described association between parkinsonism and deficiencies of complexes I and IV [11,44,45] and with the accumulation of mutations in mtDNA maintenance genes, which have been considered a risk factor for sporadic PD [13,36,46]. On the other hand, the identification of overrepresented variants in the WARS2 gene are in line with the recently reported association between biallelic mutations in this gene and early onset PD [47]. In fact, one of the two carriers reported in our cohort was diagnosed at 48 years of age.

## 5. Conclusions

In conclusion, we provide the first comprehensive genetic analysis of TFEB-targeted genes in PD patients and suggest that these genes could be involved in the pathogenesis of PD disease. On the other hand, our data replicate previous findings and expand the knowledge of the association between PD and alterations in lysosomal and mitochondrial genes. Future studies including larger case-control cohorts and functional studies will be essential to validate these findings.

## Figures and Tables

**Table 1:** Identification of variants in PD-associated genes.

Mutations Classification	Patient	Gene	Heredity	Nucleotide Change	Predicted Effect on Protein	ACMG Classification/Varsome ^1^	HGMD Classification
Monogenic-PD	25	*LRRK2*	AD	c.6566A>G	p.Tyr2189Cys	Likely benign	Disease-causing variant
	3	*PRKN*	AR	c.635G>A	p.Cys212Tyr	Likely Pathogenic	Disease-causing variant
		*PRKN*	AR	c.155delA	p.Asn52MetfsTer29	Pathogenic	Disease-causing variant
Other-PD	2	*GIGYF2*	AD	c.3167C>G	p.Ser1056Cys	VUS	Not reported
	4	*GIGYF2*	AD	c.658C>T	p.Arg220Cys	VUS	Not reported
	8	*ATXN2*	AD	c.542_543insACA	p.Gln188dup	VUS	Not reported
	10	*ATP7B*	AR	c.4301C>T	p.Thr1434Met	VUS	Dubious disease-causing variant
		*ATP7B*	AR	c.1301A>G	p.Asn434Ser	VUS	Not reported
	12	*ATXN2*	AD	c.2937 + 4A>C	p.?	VUS	Not reported
	20	*ATXN2*	AD	c.519_520delGC	p.Gln174AlafsTer75	Likely pathogenic	Not reported
	24	*DCTN1*	AD	c.2968C>T	p.Arg990Cys	VUS	Not reported
	28	*SPR*	AD; AR	c.448A>G	p.Arg150Gly	Pathogenic	Disease-causing variant
	32	*POLG*	AR	c.752C>T	p.Thr251Ile	VUS	Disease-causing variant
		*POLG*	AR	c.1760C>T	p.Pro587Leu	Likely pathogenic	Disease-causing variant

AD, Autosomal dominant; AR, Autosomal recessive; VUS, Variant of uncertain significance; Monogenic-PD, Monogenic forms of PD; Other-PD: Variants in other genes associated to PD or parkinsonism. All variants were found in heterozygosity. ^1^ https://varsome.com.

**Table 2:** Genes with overrepresented variants in PD patients.

	TFEB-Regulated Genes	Non-TFEB-Regulated Genes	Number of Genes
PD-associated genes	*-*	***ATP7B, ATXN2, C19orf12, PRKN, SPR, SYNJ1***	6
Lysosomal genes	***ATP13A2**, CD63, CTBS, **GBA**, HEXA, MPO, PS1, **SMPD1**, TMEM192, UNC13D*	*GNPTAB*	11
Mitochondrial genes	*-*	*ABCB6, CYP27A1, MFN2, MUTYH, NDUFB3, NDUFV1, **POLG**, SCO2, SLC25A46, TXNDR, WARS2*	11
Other genes	*CRY1, CSPG4, FAM83G, PSEN2, RAD9A, SQSTM1, SEMA3D, TNFAIP3*	*-*	8
Number of genes	18	18	36

**In bold**: genes previously reported to be associated to PD.

## Data Availability

Not applicable.

## Supplementary Materials

The following supporting information can be downloaded at: https://www.mdpi.com/article/10.3390/jcm11061749/s1, Figure S1: Percentage of PD patients and age-matched controls presenting the indicated number of variants in genes of each analysed group; Figure S2: Distribution of variants according to age of onset and distribution of synonymous variants. Table S1: Clinical and genetic characteristics of PD patients included in this study; Table S2: Clinical and genetic characteristics of the age-matched healthy controls included in this study. Table S3: Genes analyzed in this study.
