# Supplementary material for: Over-Mutated Mitochondrial, Lysosomal and TFEB-Regulated Genes in Parkinson’s Disease
Source: J Clin Med. 2022 Mar 21;11(6):1749. doi: 10.3390/jcm11061749 (PMC8951534; doi:10.3390/jcm11061749)
Supplement: Supplementary file 1 [file jcm-11-01749-s001.zip › Supl_Table S1.pdf]

Supplementary Table S1. Clinical and genetic characteristics of PD patients included in this study.

Patient			Clinical traits							Genetics									
**#**	**Age of onset**	**Age**	**FH**	**PH**	**MF**	**Dyskinesia**	**NMS**	**DaT-SPECT**	**Diagnosis**	**Gene**	**Gene group**	**Heredity**	**Nucleotide change**	**Predicted effect on protein**	**ACMG classification /Varsome**	**HGMD classification**	**Overrepresented**	**Reference**
**1**	48y	58y	x	x				Altered		ATP7B	PD	AR	c.3620A>G	p.(His1207Arg)	Benign	Dubious disease causing variant	Yes	Abdelghaffar (2008) J Hum Genet 53, 681
										FAM83G	TFEB		c.968C>T	p.(Pro323Leu)	Uncertain Significance	Not reported	Yes	NR
**2**	72y	79y		x						GIGYF2	PD	AD	c.3167C>G	p.(Ser1056Cys)	Uncertain Significance	Not reported	No	NR
										ACADS	Mc	AR	c.511C>T	p.(Arg171Trp)	Uncertain Significance	iv/iv functional polymorphism	No	Gregerson (1998) Hum Mol Genet 7, 619
**3**	35y	46y	x		x		x	Altered	Mixed parkinson	PRKN	PD	AR	c.635G>A	p.(Cys212Tyr)	Likely Pathogenic	Disease causing variants	Yes	Pineda-Trujillo (2001) Neurosci Lett 2, 298
										PRKN	PD	AR	c.155delA	p.(Asn52Metfs Ter29)	Pathogenic	Disease causing variants	Yes	Abbas (1999) Hum Mol Genet 8, 567
**4**	35y	66y (Age of death)			x	x			Akinetic/rigid parkinson	GIGYF2	PD	AD	c.658C>T	p.(Arg220Cys)	Uncertain Significance	Not reported	Yes	NR
										GNPTAB	LSD	AR	c.3503_3504delTC	p.(Leu1168Glnf sTer5)	Pathogenic	Disease causing variants	Yes	Kudo (2006) Am J Hum Genet 78, 451
										UNC13D	L · TFEB	AR	c.1820G>C	p.(Arg607Pro)	Likely Pathogenic	Disease causing variants	Yes	Rohr (2010) Haematologica 95, 2080
										CRY1	TFEB	AD	c.1657+3A>C		Benign	Disease causing variants	Yes	Patke (2017) Cell 169, 203
										CSPG4	TFEB		c.3097G>A	p.(Gly1033Arg)	Uncertain Significance	Not reported	Yes	NR
**5**	77y	83y	x	x			x		Mixed parkinson	GAA	LSD · TFEB	AR	c.-32-13T>G		Pathogenic	Disease causing variants	No	Huie (1994) Hum Mol Genet 3, 2231
										PSEN2	TFEB	AD	c.389C>T	p.(Ser130Leu)	Benign	Dubious disease causing variant	Yes	Tedde (2003) Arch Neurol 60, 1541
**6**	72y	86y (Age of death)					x	Altered	Akinetic/rigid parkinson	GNPTAB	LSD	AR	c.1433T>C	p.(Ile478Thr)	Uncertain Significance	Dubious disease causing variant	Yes	Raza (2016) Eur J Hum Genet 24, 529
										MPO	L · TFEB	AR	c.1705C>T	p.(Arg569Trp)	Pathogenic	Disease causing variants	No	Nauseef (1994) J Biol Chem 269, 1212
										MUTYH	Mc	AR	c.536A>G	p.(Tyr179Cys)	Likely Pathogenic	Disease causing variants	Yes	Al-Tassan (2002) Nat Genet 30, 227
										OGG1	Mc	?	c.923G>A	p.(Gly308Glu)	Uncertain Significance	Dubious disease causing variant	No	Morak (2011) Eur J Cancer 47, 1046
**7**	66y	81y		x			x		Mixed parkinson	ATP7B	PD	AR	c.1934T>G	p.(Met645Arg)	Likely Pathogenic	Disease causing	Yes	Shah (1997) Am J Hum Genet 61, 317

															variants		
									SEMA3D	TFEB		c.170G>T	p.(Ser57Ile)	Uncertain Significance	Not reported	*Not present in Gnomad*	NR
**8**	54y	68y	x	x			x	Mixed parkinson	ATXN2	PD	AD	c.542_543insACA	p.(Gln188dup)	Uncertain Significance	Not reported	*Not present in Gnomad*	NR
									CTBS	L · TFEB		c.648G>T	p.(Trp216Cys)	Uncertain Significance	Not reported	Yes	NR
									TXNRD2	Mc	AR	c.1341T>G	p.(Tyr447Ter)	Pathogenic	Disease causing variants	Yes	Prasad (2014) J Clin Endocrinol Metab 99, E1556
**9**	48y	54y	x		x		x	Akinetic/rigid parkinson	CYP27A1	Mc	AR	c.1436G>A	p.(Arg479His)	Likely Pathogenic	Not reported	Yes	NR
**10**	76y	80y	x	x		x		Tremoric parkinson	ATP7B	PD	AR	c.4301C>T	p.(Thr1434Met)	Uncertain Significance	Dubious disease causing variant	Yes	Loudianos (1999) J Med Genet 36, 833
									ATP7B	PD	AR	c.1301A>G	p.(Asn434Ser)	Uncertain Significance	Not reported	Yes	NR
									GNPTAB	LSD	AR	c.1433T>C	p.(Ile478Thr)	Uncertain Significance	Dubious disease causing variant	Yes	Raza (2016) Eur J Hum Genet 24, 529
									GBA	LSD · TFEB	AR	c.-15A>G		Uncertain Significance	Dubious disease causing variant	No	Orme (2020) Acta Neuropathol Commun 8, 5
									HPS1	L · TFEB	AR	c.1132A>G	p.(Ile378Val)	Likely Benign	Not reported	*Not present in Gnomad*	NR
									CRY1	TFEB	AD	c.1657+3A>C		Benign	Disease causing variants	Yes	Patke (2017) Cell 169, 203
**11**	66y	71y		x				Mixed parkinson	ATP7B	PD	AR	c.4135C>T	p.(Pro1379Ser)	Pathogenic	Disease causing variants	No	Cox (2005) Hum Mutat 26, 280
									PRKN	PD	AR	c.1180G>A	p.(Asp394Asn)	Benign	iv/iv functional polymorphism	No	Lucking (2003) Arch Neurol 60, 1253
									NDUFB3	Mc	AR	c.64T>C	p.(Trp22Arg)	Pathogenic	Disease causing variants	Yes	Calvo (2012) Sci Transl Med 4, 118ra10
									COQ8B	Mc	AR	c.187C>T	p.(Arg63Trp)	Uncertain Significance	Dubious disease causing variant	No	Landis (2017) J Cardiovasc Transl Res 10, 423
**12**	47y	69y (Age of death)			x	x	x	Akinetic/rigid parkinson	PRKN	PD	AR	c.1310C>T	p.(Pro437Leu)	Likely Pathogenic	Disease causing variants	No	Foroud (2003) Neurology 60, 796
									SYNJ1	PD	AR	c.4033G>A	p.(Val1345Ile)	Likely Benign	Dubious disease causing variant	Yes	Bandrés-Ciga (2016) Neurobiol Aging 45, 213.e3
									ATXN2	PD	AD	c.2937+4A>C		Uncertain Significance	Not reported	Yes	NR
									CYP27A1	Mc	AR	c.1151C>T	p.(Pro384Leu)	Benign	Dubious disease causing variant	No	Verrips (2000) Brain 123, 908
**13**	64y	71y	x					Tremoric parkinson	SMPD1	LSD · TFEB	AR	c.1550A>T	p.(Glu517Val)	Likely Pathogenic	Disease causing variants	No	Simonaro (2002) Am J Hum Genet 71, 1413
									ACO2	Mc	AR	c.220C>G	p.(Leu74Val)	VUS	Disease causing variants	No	Metodiev (2014) J Med Genet 51, 834

**14**	73y	85y		x	x	x			Akinetic/rigid parkinson	GAA	LSD · TFEB	AR	c.271G>A	p.(Asp91Asn)	Benign	Dubious disease causing variant	No	Labrousse (2010) Mol Genet Metab99, 379
**15**	35y	41y		x	x		x	altered	Mixed parkinson	ATP13A2	LSD · TFEB	AR	c.238T>C	p.(Cys80Arg)	VUS	Not reported	Yes	NR
										GAA	LSD · TFEB	AR	c.271G>A	p.(Asp91Asn)	Benign	Dubious disease causing variant	No	Labrousse (2010) Mol Genet Metab99, 379
										CTBS	L	?	c.724A>T	p.(Ile242Phe)	VUS	Not reported	Yes	
**16**	42y	55y		x	x	x			Mixed parkinson	FAM83G	TFEB		c.1888C>T	p.(Arg630Trp)	Likely Benign	iv/iv functional polymorphism	No	Loomis (2019) Sci Rep 9, 5942
**17**	76y	82y	x						Akinetic/rigid parkinson	TMEM192	L · TFEB		c.371G>A	p.(Arg124Gln)	VUS	Not reported	Yes	NR
										CSPG4	TFEB		c.2702T>G	p.(Val901Gly)	Likely Benign	Dubious disease causing variant	Yes	de Vrij (2019) Mol Psychiatry 24, 757
										ABCB6	Mc	AD	c.2168G>A	p.(Arg723Gln)	Pathogenic	Disease causing variants	Yes	Bawazir (2014) Transfusion 54, 3043
**18**	74y	79y	x	x			x	altered	Tremoric parkinson	ATP7B	PD	AR	c.3620A>G	p.(His1207Arg)	Benign	Dubious disease causing variant	Yes	Abdelghaffar (2008) J Hum Genet 53, 681
**19**	63y	68y	x	x			x		Akinetic/rigid parkinson	SMPD1	LSD · TFEB	AR	c.106G>T	p.(Val36Leu)	Uncertain Significance	Not reported	Yes	NR
										HPS1	L · TFEB	AR	c.1915G>A	p.(Gly639Ser)	Uncertain Significance	Dubious disease causing variant	Yes	Stearman (2019) Am J Respir Crit Care Med epub, epub
**20**	42y	60y	x		x	x	x			ATXN2	PD	AD	c.519_520delGC	p.(Gln174Alafs Ter75)	Likely Pathogenic	Not reported	Yes	NR
										SMPD1	LSD · TFEB	AR	c.1022G>C	p.(Arg341Pro)	Likely Pathogenic	Not reported	Yes	NR
										SEMA3D	TFEB		c.1843C>A	p.(Pro615Thr)	Likely Benign	Dubious disease causing variant	No	Jiang (2012) Hum Mutat 33, 281
										SLC25A46	Mc	AR	c.1018C>T	p.(Arg340Cys)	Pathogenic	Disease causing variants	Yes	Abrams (2015) Nat Genet 47, 926
										NDUFV1	Mc	AR	c.1162+4A>C		Likely pathogenic	Disease causing variants	Yes	Benit (2001) Am J Hum Genet 68, 1344
**21**	48y	55y		x				altered	Mixed parkinson	PRKN	PD	AR	c.1180G>A	p.(Asp394Asn)	Benign	iv/iv functional polymorphism	No	Lucking (2003) Arch Neurol 60, 1253
										GBA	LSD · TFEB	AR	c.1300C>T	p.(Arg434Cys)	Likely Pathogenic	Disease causing variants	Yes	Rozenberg (2006) Blood Cells Mol Dis 37, 204
										HEXA	LSD · TFEB	AR	c.1073+1G>T		Pathogenic	Disease causing variants	Yes	Akli (1991) Genomics 11, 124
										SQSTM1	TFEB	AD; AR	c.1175C>T	p.(Pro392Leu)	VUS	Disease causing variants	Yes	Laurin (2002) Am J Hum Genet 70, 1582
										TGM5	TFEB	AR	c.337G>T	p.(Gly113Cys)	Likely Pathogenic	Disease causing variants	Yes	Cassidy (2005) Am J Hum Genet 77, 909
										MUTYH	Mc	AR	c.536A>G	p.(Tyr179Cys)	Likely Pathogenic	Disease causing	Yes	Al-Tassan (2002) Nat Genet 30, 227

															variants		
										WARS2	Mc	AR	c.37T>G	p.(Trp13Gly)	Benign	Disease causing variants	Yes	Musante (2017) Hum Mutat 38, 621
**22**	82y	86y	x				x		Mixed parkinson	SCO2	Mc	AD	c.341G>A	p.(Arg114His)	Pathogenic	Disease causing variants	Yes	Tran-Viet (2013) Am J Hum Genet 92, 820
										POLG	Mc	AR	c.1550G>T	p.(Gly517Val)	Benign	Dubious disease causing variant	No	Horvath (2006) Brain 129, 1674
**23**	72y	80y						altered	Mixed parkinson	TRAP1	Mc	?	c.383G>A	p.(Arg128His)	Likely benign	Disease causing variants	No	Omoyinmi (2017) PLoS One 12, e0181874
										ACADS	Mc	AR	c.511C>T	p.(Arg171Trp)	Uncertain Significance	iv/iv functional polymorphism	No	Gregerson (1998) Hum Mol Genet 7, 619
										UCP3	Mc	AD; AR	c.304G>A	p.(Val102Ile)	Benign	iv/iv functional polymorphism	*Not present in Gnomad*	Adams (2009) J Nutr 139, 1073
**24**	35y	41y			x	x	x		Mixed parkinson	ATP7B	PD	AR	c.3620A>G	p.(His1207Arg)	Benign	Dubious disease causing variant	Yes	Abdelghaffar (2008) J Hum Genet 53, 681
										DCTN1	PD	AD	c.2968C>T	p.(Arg990Cys)	Uncertain Significance	Not reported	Yes	NR
**25**	40y	57y	x	x	x	x	x		Mixed parkinson	LRRK2	PD	AD	c.6566A>G	p.(Tyr2189Cys)	Likely benign	Disease causing variants	Yes	Nuytemans (2008) Eur J Hum Genet 16, 471
										GBA	LSD · TFEB	AR	c.1223C>T	p.(Thr408Met)	Uncertain Significance	Dubious disease causing variant	No	Beutler (1996) Proc Assoc Am Physicians 108, 179
										GBA	LSD · TFEB	AR	c.1000-15G>A		Uncertain Significance	Not reported	*Not present in Gnomad*	NR
										RAD9A	L		c.716A>G	p.(His239Arg)	Likely benign	iv/iv functional polymorphism	Yes	Maniwa (2006) Cancer 106, 1117
										TCIRG1	Mc	AR	c.1249G>A	p.(Ala417Thr)	Likely pathogenic	Disease causing variants	No	Pangrazio (2012) Osteoporos Int 23, 2713
**26**	45y	58y			x	x	x	altered	Mixed parkinson	ACADS	Mc	AR	c.511C>T	p.(Arg171Trp)	Uncertain Significance	iv/iv functional polymorphism	No	Gregerson (1998) Hum Mol Genet 7, 619
**27**	62y	70y	x			x			Mixed parkinson	GBA	LSD · TFEB	AR	c.1300C>T	p.(Arg434Cys)	Likely Pathogenic	Disease causing variants	Yes	Rozenberg (2006) Blood Cells Mol Dis 37, 204
										TGM5	TFEB	AR	c.337G>T	p.(Gly113Cys)	Likely Pathogenic	Disease causing variants	Yes	Cassidy (2005) Am J Hum Genet 77, 909
										MUTYH	Mc	AR	c.536A>G	p.(Tyr179Cys)	Likely Pathogenic	Disease causing variants	Yes	Al-Tassan (2002) Nat Genet 30, 227
										WARS2	Mc	AR	c.37T>G	p.(Trp13Gly)	Benign	Disease causing variants	Yes	Musante (2017) Hum Mutat 38, 621
										ELAC2	Mc	AR	c.1621G>A	p.(Ala541Thr)	Uncertain Significance	iv/iv functional polymorphism	No	Rebbeck (2000) Am J Hum Genet 67, 1014
**28**	53y	62y		x			x			SPR	PD · Mc	AD	c.448A>G	p.(Arg150Gly)	Pathogenic	Disease causing variants	Yes	Bonafe (2001) Am J Hum Genet 69, 269
										SYNJ1	PD	AR	c.1147G>A	p.(Ala383Thr)	Likely benign	Dubious disease	Yes	Bandrés-Ciga (2016) Neurobiol

															causing variant		Aging 45, 213.e3
									GBA	LSD · TFEB	AR	c.1342G>C	p.(Asp448His)	Uncertain Significance	Disease causing variants	Yes	Eyal (1990) Gene 96, 277
									SQSTM1	TFEB	AD; AR	c.1175C>T	p.(Pro392Leu)	VUS	Disease causing variants	Yes	Laurin (2002) Am J Hum Genet 70, 1582
**29**	33y	42y	x		x		x	Mixed parkinson	MFN2	Mc	AD; AR	c.2258dupT	p.(Gln754Alafs Ter9)	Pathogenic	Disease causing variants	Yes	Engelfried (2006) BMC Med Genet 7, 53
**30**	60y	66y						Tremoric parkinson	ACADM	Mc	AR	c.734C>T	p.(Ser245Leu)	Pathogenic	Disease causing variants	*Not present in Gnomad*	Zschocke (2001) Hum Genet 108, 404
									POLG	Mc	AR	c.1550G>T	p.(Gly517Val)	Benign	Dubious disease causing variant	No	Horvath (2006) Brain 129, 1674
									FPGS	Mc	?	c.1396C>T	p.(Arg466Cys)	Likely benign	iv/iv functional polymorphism	Yes	Leil (2007) Cancer Res 67, 8772
**31**	38y	43y	x	x			x		C19orf12	PD		c.346G>A	p.(Val116Met)	Uncertain Significance	Not reported	No	NR
									GNPTAB	LSD	AR	c.512C>T	p.(Ala171Val)	Uncertain Significance	Not reported	Yes	NR
									DGUOK	Mc	AR	c.509A>G	p.(Gln170Arg)	Benign	Dubious disease causing variant	No	Freisinger (2006) Arch Neurol 63, 1129
**32**	39y	48y	x		x	x			ACAD9	Mc	AR	c.976G>A	p.(Ala326Thr)	Benign	Dubious disease causing variant	No	Ilinca (2020) Stroke 51, 1056
									POLG	Mc	AR	c.752C>T	p.(Thr251Ile)	VUS	Disease causing variants	No	Lamantea (2002) Ann Neurol 52, 211
									POLG	Mc	AR	c.1760C>T	p.(Pro587Leu)	Likely pathogenic	Disease causing variants	No	Van Goethem (2003) Eur J Hum Genet 11, 547
									CYP11A1	Mc	?	c.940G>A	p.(Glu314Lys)	Pathogenic	Disease causing variants	No	Chan (2015) Front Endocrinol (Lausanne) 6, 113
									CHCHD10	Mc	AD	c.100C>T	p.(Pro34Ser)	Benign	Dubious disease causing variant	No	Chaussenot (2014) Neurobiol Aging 35, 2884.e1

All the variants were found in heterozygosity. FH: Family history; PH: Pathological history; FM: Motor fluctuations; NMS: Non-motor symptoms; PD: Parkinson disease; Mc: Mitochondrial function; L: Lysosomal; LSD: Lysosomal storage disease; AR: Autosomal recessive; AD: Autosomal dominant; VUS: Variant of Unknown Significance; iv/iv: In vitro/in vivo; NR: Non reported. Overrepresented when comparing the variant frequency in PD patients and the frequency in GnomAD database, and reference if it has been reported before.
