# Supplementary material for: Over-Mutated Mitochondrial, Lysosomal and TFEB-Regulated Genes in Parkinson’s Disease
Source: J Clin Med. 2022 Mar 21;11(6):1749. doi: 10.3390/jcm11061749 (PMC8951534; doi:10.3390/jcm11061749)
Supplement: Supplementary file 1 [file jcm-11-01749-s001.zip › Supl_Table S2.pdf]

Supplementary Table S2. Clinical and genetic characteristics of the age-matched healthy controls included in this study.

	Genetics							
**Number**	**Gene**	**Gene group**	**Heredity**	**Nucleotide change**	**Predicted effect on protein**	**ACMG classification/Varsome**	**HGMD classification**	**Reference**
**C1**	PRKN	PD	AR	c.1204C>T	p.(Arg402Cys)	Benign	Dubious disease causing variant	Bertoli-Avella (2005) Mov Disord 20, 424
	SEMA3D	TFEB	AR	c.335A>T	p.(Glu112Val)	Uncertain Significance	Not reported	NR
	TRAP1	Mc		c.1330T>A	p.(Tyr444Asn)	Likely Benign	Dubious disease causing variant	Saisawat (2014) Kidney Int 85, 1310
**C2**	ATP7B	PD	AR	c.4301C>T	p.(Thr1434Met)	Uncertain Significance	Dubious disease causing variant	Loudianos (1999) J Med Genet 36, 833
	GALNS	LSD · TFEB	AR	c.1145G>A	p.(Arg382Gln)	Pathogenic	Not reported	NR
**C3**	*No variants*							
**C4**	*No variants*							
**C5**	GBA	LSD · TFEB	AR	c.1093G>A	p.(Glu365Lys)	Benign	Dubious disease causing	Eyal (1991) Hum Genet 87, 328
	FOLR1	TFEB	AR	c.610C>T	p.(Arg204Ter)	Pathogenic	Disease causing variant	Dill (2011) Mol Genet Metab 104, 362
**C6**	ATP7B	PD	AR	c.3620A>G	p.(His1207Arg)	Benign	Dubious disease causing variant	Abdelghaffar (2008) J Hum Genet 53, 681
**C7**	GBA	LSD · TFEB	AR	c.1223C>T	p.(Thr408Met)	Uncertain Significance	Dubious disease causing variant	Beutler (1996) Proc Assoc Am Physicians 108, 179
	ACADS	Mc	AR	c.1156C>T	p.(Arg386Cys)	Uncertain Significance	Disease causing variant	Merinero (2006) J Inherit Metab Dis 29, 685
**C8**	HGSNAT	LSD	AR	c.1843G>A	p.(Ala615Thr)	Benign	Disease causing variant	Hrebicek (2006) Am J Hum Genet 79, 807
**C9**	HSPA6	TFEB		c.1340G>T	p.(Gly447Val)	NR	Disease causing variant	Kause (2019) Birth Defects Res 111, 591

**C10**	GBA	LSD · TFEB	AR	c.1223C>T	p.(Thr408Met)	Uncertain Significance	Dubious disease causing variant	Beutler (1996) Proc Assoc Am Physicians 108, 179
	GNPTAB	LSD	AR	c.3503_3504delTC	p.(Leu1168GlnfsTer5)	Pathogenic	Disease causing variant	Kudo (2006) Am J Hum Genet 78, 451
	IDUA	LSD · TFEB	AR	c.246C>G	p.(His82Gln)	Uncertain Significance	Dubious disease causing variant	Scott (2013) J Pediatr 163, 498
	PSEN2	TFEB	AR	c.554A>G	p.(Tyr185Cys)	Uncertain Significance	Not reported	NR
**C11**	ACAD9	Mc	AR	c.976G>A	p.(Ala326Thr)	Benign	Dubious disease causing variant	Ilinca (2020) Stroke 51, 1056
**C12**	MUTYH	Mc	AR	c.1187G>A	p.(Gly396Asp)	Pathogenic	Disease causing variant	Al-Tassan (2002) Nat Genet 30, 227
	SCO2	Mc	AR;AD	c.341G>A	p.(Arg114His)	Pathogenic	Disease causing variant	Tran-Viet (2013) Am J Hum Genet 92, 820
	CRYAB	TFEB	AR	c.343delT	p.(Ser115ProfsTer14)	Pathogenic	Disease causing variant	Forrest (2011) Neuromuscul Disord 21, 37
**C13**	PRKN	PD	AR	c.1180G>A	p.(Asp394Asn)	Benign	iv/iv functional polymorphism	Lucking (2003) Arch Neurol 60, 1253
	ATP7B	PD	AR	c.3688A>G	p.(Ile1230Val)	Pathogenic	Disease causing variant	Davies (2008) Genet Test 12, 139
	HTRA2	PD	AR	c.421G>T	p.(Ala141Ser)	Benign	iv/iv functional polymorphism	Strauss (2005) Hum Mol Genet 14, 2099
	TRAP1	Mc		c.1406G>A	p.(Arg469His)	Uncertain Significance	Disease causing variant	Saisawat (2014) Kidney Int 85, 1310
**C14**	MAN2B1	LSD · TFEB	AR	c.844C>T	p.(Pro282Ser)	Likely pathogenic	Disease causing variant	Matlach (2018) Orphanet J Rare Dis 13, 88
**C15**	SPG7	Mc	AD; AR	c.1529C>T	p.(Ala510Val)	Pathogenic	Disease causing variant	Brugman (2008) Neurology 71, 1500
	DLST	Mc	AD; AR	c.1121G>A	p.(Gly374Glu)	Likely pathogenic	Disease causing variant	Remacha (2019) Am J Hum Genet 104, 651
**C16**	LIPA	LSD · TFEB	AR	c.894G>A	p.(Gln298=)	Pathogenic	Disease causing variant	Scott (2013) Hepatology 58, 958

	MRPL3	Mc	AR	c.862T>C	p.(Ser288Pro)	Uncertain Significance	Dubious disease causing variant	Neubauer (2017) Eur J Hum Genet 25, 404
**C17**	HEXB	LSD · TFEB	AR	c.1250C>T	p.(Pro417Leu)	Pathogenic	Disease causing variant	Gomez-Lira (1995) Hum Genet 96, 417
	PKLR	Mc	AR	c.1456C>T	p.(Arg486Trp)	Pathogenic	Disease causing variant	Baronciani (1993) Proc Natl Acad Sci U S A 90, 4324
	CRYAB	TFEB	AR	c.470G>A	p.(Arg157His)	Benign	Disease causing variant	Inagaki (2006) Biochem Biophys Res Commun 342, 379
**C18**	PRKN	PD	AR	c.1180G>A	p.(Asp394Asn)	Benign	iv/iv functional polymorphism	Lucking (2003) Arch Neurol 60, 1253
**C19**	GBA	LSD · TFEB	AR	c.1342G>C	p.(Asp448His)	Uncertain Significance	Disease causing variant	Eyal (1990) Gene 96, 277
	LRRK2	PD	AR	c.2769G>C	p.(Gln923His)	Uncertain Significance	Dubious disease causing variant	Camargos (2009) Mov Disord 24, 662
	ACADS	Mc	AR	c.511C>T	p.(Arg171Trp)	Uncertain Significance	iv/iv functional polymorphism	Gregerson (1998) Hum Mol Genet 7, 619
**C20**	CSPG4	TFEB	AR	c.5156A>G	p.(Gln1719Arg)	Uncertain Significance	Not reported	NR
**C21**	LAMP2	PD	AR	c.661G>A	p.(Gly221Arg)	Benign	Dubious disease causing variant	Mook (2013) J Med Genet 50, 614
	ACADS	Mc	AR	c.511C>T	p.(Arg171Trp)	Uncertain Significance	iv/iv functional polymorphism	Gregerson (1998) Hum Mol Genet 7, 619
	TACC2	TFEB	AR	c.8188G>A	p.(Gly2730Arg)	Benign	Dubious disease causing variant	Bruse (2016) Hum Genomics 10, 1
**C22**	ARSA	LSD · TFEB	AR	c.585G>T	p.(Trp195Cys)	Benign	iv/iv functional polymorphism	Ricketts (1996) J Affect Disord 40, 137
	LRRK2	PD	AR	c.6241A>G	p.(Asn2081Asp)	Benign	iv/iv functional polymorphism	Heckman (2014) Neurology 83, 2256
	ICAM1	TFEB		c.1055C>T	p.(Pro352Leu)	NR	iv/iv functional polymorphism	Vischer (2008) Pharmacogenet Genomics 18, 1017
**C23**	CLN6	LSD	AR	c.486+8C>T		Benign	Dubious disease causing variant	Kousi (2012) Hum Mutat 33, 42

	HTRA2	PD	AR	c.421G>T	p.(Ala141Ser)	Benign	iv/iv functional polymorphism	Strauss (2005) Hum Mol Genet 14, 2099
	LRRK2	PD	AR	c.4937T>C	p.(Met1646Thr)	Benign	iv/iv functional polymorphism	Ross (2011) Lancet Neurol 10, 898
**C24**	ATXN2	PD	AD	c.540delG	p.(Gln180HisfsTer26)	Likely pathogenic	Not reported	NR
	ATXN2	PD	AD	c.534_538delGCAAC	p.(Gln179AlafsTer69)	Pathogenic	Not reported	NR
	ATP7B	PD	AR	c.3889G>A	p.(Val1297Ile)	Benign	Dubious disease causing variant	Li (2011) BMC Med Genet 12, 6
	GALNS	LSD · TFEB	AR	c.517T>G	p.(Phe173Val)	Pathogenic	Not reported	NR
	MPO	L	AR	c.752T>C	p.(Met251Thr)	Likely pathogenic	Dubious disease causing variant	Romano (1997) Blood 90, 4126
	MUTYH	Mc	AR	c.1187G>A	p.(Gly396Asp)	Pathogenic	Disease causing variant	Al-Tassan (2002) Nat Genet 30, 227
**C25**	*No variants*							
**C26**	HPS5	L	AR	c.3293C>T	p.(Thr1098Ile)	Benign	Dubious disease causing variant	Huizing (2004) Traffic 5, 711
	HOGA1	Mc	Ar	c.700+5G>T		Pathogenic	Disease causing variant	Belostotsky (2010) Am J Hum Genet 87, 392
**C27**	PRKN	PD	AR	c.1180G>A	p.(Asp394Asn)	Benign	iv/iv functional polymorphism	Lucking (2003) Arch Neurol 60, 1253
	GLA	LSD · TFEB	XLR	c.937G>T	p.(Asp313Tyr)	Pathogenic	Dubious disease causing variant	Eng (1993) Am J Hum Genet 53, 1186
	GLDC	Mc	AR	c.1705G>A	p.(Ala569Thr)	Benign	Disease causing variant	Kure (2006) Hum Mutat 27, 343
**C28**	ATP7B	PD	AR	c.4135C>T	p.(Pro1379Ser)	Pathogenic	Disease causing variant	Cox (2005) Hum Mutat 26, 280
	ARSA	LSD · TFEB	AR	c.869G>A	p.(Arg290His)	Pathogenic	Disease causing variant	Gort (1999) Hum Mutat 14, 240
	SPINK1	TFEB	AR	c.163C>T	p.(Pro55Ser)	Benign	Dubious disease causing variant	Chen (2001) Gastroenterology 120, 1061

**C29**	PRKN	PD	AR	c.574A>C	p.(Met192Leu)	Benign	Dubious disease causing variant	Hedrich (2002) Neurology 58, 1239
	HGSNAT	LSD	AR	c.1880A>G	p.(Tyr627Cys)	Uncertain Significance	Disease causing variant	Ouesleti (2011) Clin Chim Acta 412, 2326
	IDUA	LSD · TFEB	AR	c.246C>G	p.(His82Gln)	Uncertain Significance	Dubious disease causing variant	Scott (2013) J Pediatr 163, 498
**C30**	GBA	LSD · TFEB	AR	c.1223C>T	p.(Thr408Met)	Uncertain Significance	Dubious disease causing variant	Beutler (1996) Proc Assoc Am Physicians 108, 179
	ATP7B	PD	AR	c.3620A>G	p.(His1207Arg)	Benign	Dubious disease causing variant	Abdelghaffar (2008) J Hum Genet 53, 681
	FAM83G	TFEB	AR	c.1888C>T	p.(Arg630Trp)	Likely Benign	iv/iv functional polymorphism	Loomis (2019) Sci Rep 9, 5942
	TGM5	TFEB	AR	c.337G>T	p.(Gly113Cys)	Pathogenic	Disease causing variant	Cassidy (2005) Am J Hum Genet 77, 909

All the variants were found in heterozygosity. PD: Parkinson disease; Mc: Mitochondrial function; L: Lysosomal; LSD: Lysosomal storage disease; AR: Autosomal recessive; AD: Autosomal dominant; NR: Non reported.
