# Supplementary material for: Over-Mutated Mitochondrial, Lysosomal and TFEB-Regulated Genes in Parkinson’s Disease
Source: J Clin Med. 2022 Mar 21;11(6):1749. doi: 10.3390/jcm11061749 (PMC8951534; doi:10.3390/jcm11061749)
Supplement: Supplementary file 1 [file jcm-11-01749-s001.zip › Supl_Table S3.pdf]

Supplementary Table S3. Genes analyzed in this study.

**Lysosomal genes** (138 genes)	**Genes associated to lysosomal storage diseases** (56 genes)	AGA, ARSA, ARSB, ASAH1, CLN3, CLN5, CLN6, CLN8, CTNS, CTSA, CTSD, CTSF, CTSK, FUCA1, GAA, GALC, GALNS, GBA, GLA, GLB1, GM2A, GNE, GNPTAB, GNPTG, GNS, GUSB, HEXA, HEXB, HGSNAT, HSPG2, HYAL1, IDS, IDUA, LAMP2, LIPA, MAN2B1, MANBA, MCOLN1, MFSD8, NAGA, NAGLU, NEU1, NPC1, NPC2, NPL, PPT1, PSAP, SCARB2, SGSH, SIAE, SLC17A5, SMPD1, ST3GAL5, STS, SUMF1, TPP1.
	**Genes not yet associated to lysosomal storage diseases** (82 genes)	ABCA2, ABCB9, ACP2, ACP5, AP3B1, AP3D1, ARSG, BLOS3, BLOS6, C1orf85, C2orf18, C7orf28A, CD63, CD68, CHS1, CLCN7, CPVL, CREG1, CTBS, CTSB, CTSC, CTSH, CTSL, CTSS, CTSZ, DNASE2, DTNBP1, ENTPD4, EPDR1, EPG5, GGH, HPS1, HPS2, HPS3, HPS4, HPS5, HPS6, HPS8, HPS9, HPSE, HYAL2, IFI30, LAMP1, LAMP3, LAMTOR2, LAPTM4A, LAPTM4B, LAPTM5, LGMN, LITAF, LMBRD1, LYST, MAN2B2, MLPH, MPO, MYO5A, NBEAL2, NCSTN, NEU4, OSTM1, PA, PCYOX1, PLBD2, PPT2, RAB27A, RABGGTA, RNASET2, SCPEP1, SLC36A1, STX11, STXBP2, TMEM175, TMEM192, TMEM55B, TMEM74, TMEM9, TMEM92, TSPAN1, UNC-13, UNC13D, VIPAR, VPS33B.
**TFEB- regulated genes** (428 genes)		ABCA2, ABCB9, ACBD3, ACP5, ADFP, ADM, AHNAK2, ALOXE3, AMPD3, ANKRD1, AP1S1, AP1S2, AP4M1, APBB3, ARAP3, ARL8A, ARSA, ARSB, ARSD, ARSG, ASAH1, ATG14, ATG2, ATG4, ATG4D, ATG5, ATG7, ATG8, ATG9B, ATP13A2, ATP6AP1, ATP6V0A1, ATP6V0B, ATP6V0C, ATP6V0D1, ATP6V0D2, ATP6V0E1, ATP6V1A, ATP6V1B2, ATP6V1C1, ATP6V1D, ATP6V1E1, ATP6V1G1, ATP6V1H, AVPI1, AXUD1, BCL2, BECN1, BHLHB2, BHLHB3, BIRC3, BLOC1S1, BLOC1S3, BLVRB, BRI3, C12orf49, C17orf80, C1orf54, C1orf85, C1R, C2orf18, C5orf24, C7orf20, CABLES1, CAMKK1, CCDC43, CCRL2, CD164, CD22, CD300C, CD63, CD68, CDKN1A, CEP250, CEP72, CFB, CHKA, CHRM4, CHUK, CLC, CLC7, CLCN5, CLCN6, CLCN7, CLEC17A, CLN3, CLN5, CLTA, CLTC, CPA2, CPA4, CPVL, CRY1, CRYAB, CSF1R, CSPG4, CSTB, CTBS, CTNS, CTSA, CTSC, CTSD, CTSF, CTSH, CTSK, CTSL, CTSS, CTSZ, CXCL16, CYB5R1, DAB2, DCPS, DDI2, DDX58, DENND2D, DEXI, DNAJB4, DNAJC13, DNASE2, DUSP10, DVL2, EIF2C4, ELOVL7, EPS15L1, EPS8, ESCO1, FAM102A, FAM124A, FAM21A, FAM21B, FAM27E3, FAM54A, FAM83G, FBXO32, FLJ41484, FNBP1L, FNIP2, FOLR1, FUCA2, GAA, GABARAP, GALC, GALNS, GBA, GDF15, GEM, GGA2, GGH, GIPR, GLA, GLB1, GM2A, GNB2, GNPDA1, GNPTG, GNS, GPLD1, GPR175, GPR56, GRAMD1B, GRN, GSR, GSTO1, GTF2H1, GUSB, HES1, HEXA, HEXB, HEY1, HIF1A, HK2, HKDC1, HLA-B, HMOX1, HOXB9, HPS1, HPS3, HPS5, HPSE, HSPA1A, HSPA1L, HSPA2, HSPA5, HSPA6, HSPA8, HSPB8, HSPBAP1, HSPC159, HTRA2, HYAL2, ICAM1, IDUA, IFI30, IGF2R, IL16, IL6R, INSIG1, ITPKB, JPH1, KCNAB2, KCNK9, KCNN4, KIAA0415, KIAA1632, KIAA1737, KIFC3, LAMA1, LAMB3, LAMP1, LAMTOR1, LAPTM4A, LCE2C, LCN8, LIF, LINS1, LIPA, LOC100128975, LOC100132929, LOC201164, LOC440957, LOC643338, LOC644714, LOC646993, LOC654433, LPAR5, LRRC8B, LSS, LY6K, LYPD3, LYPD5, LYPLA3, M6PR, MAFF, MAN2B1, MANBA, MAP1LC3B, MAP3K13, MARCH8, MCCC1, MCOLN1, MFSD1, MFSD8, MMP12, MPHOSPH10, MPO, MTMR10, NAGA, NAGK, NAGLU, NAGPA, NAPA, NBR1, NDRG1, NEDD4L, NEU1, NEU4, NHEDC2, NIT1, NPC1, NPC2, NR1D1, NR1D2, NRBF2, NSMCE2, NUFIP1, NUP50, p53, P76, PCGF1, PDE2A, PDP2, PDXDC2, PEA15, PER3, PFKFB3, PIM1, PIP4K2C, PLBD2, PLCD1, PLCXD1, PLD3, PLEKHM1, PLK3, PMP22, PPARGC1A, PPIF, PPP1R13B, PPT1, PRKAG2, PSAP, PSEN2, PSG4, PTGES, PTP4A3, PTTG1IP, RAB17, RAB3IL1, RAB7A, RAB9A, RAD9A, RAET1E, RASD2, RASGRP3, RCSD1, RGS16, RLTPR, RNASET2, RPP25, RRAGA, RRAGB, RRAGC, RRAGD, RREB1, RYBP, SBNO2, SCARB2, SCPEP1, SECTM1, SELRC1, SEMA3D, SERTAD2, SETD4, SETDB2, SFXN3, SGSH, SH3BP2, SH3RF2, SLAMF7, SLC12A4, SLC16A6, SLC17A5, SLC19A2, SLC20A1, SLC25A16, SLC25A22, SLC25A25, SLC25A40, SLC26A11, SLC26A9, SLC29A1, SLC2A1, SLC36A1, SLC37A3, SLCO4A1, SMCR8, SMPD1, SNAI2, SNX16, SNX27, SNX8, SPG21, SPINK1, SPNS1, SPTBN1, SQSTM1, SRRD, ST3GAL1, ST3GAL4, STK17B, STK19, STK4, STOM, STS, STX4, STX6, SULT1C2, SUMF1, SUNC1, SUV39H1, SYNJ2, TACC2, TAF4B, TAX1BP1,

		TBC1D14, TCTEX1D2, TEAD3, TGM5, TIAF1, TM4SF19, TMCC3, TMEFF1, TMEM159, TMEM16B, TMEM192, TMEM38B, TMEM55B, TMEM80, TNFAIP3, TOB1, TOM1, TPP1, TRAF5, TRAPPC2L, TRIM63, TRPM7, UAP1L1, UBA52, UCA1, UCK1, UGDH, UNC13D, UPP1, UST, UVRAG, VAC14, VAMP8, VASN, VEGFA, VPS11, VPS16, VPS18, VPS26A, VPS33A, VPS35, VPS41, VPS8, WDR45, WDR81, WDTC1, WIPI1, XRCC2, ZCCHC8, ZDHHC3, ZFYVE26, ZNF330, ZNF57, ZSCAN5A.
**Genes associated to Parkinson disease** (53 genes)		ATP13A2, ATP1A3, ATP6AP2, ATP7B, C19orf12, DCTN1, DNAJC12, DNAJC13, DNAJC5, DNAJC6, FBXO7, FTL, FUS, GCH1, GIGYF2, GRN, HTRA2, KCTD7, LRRK2, MAPT, PANK2, PARK10, PARK12, PARK16, PARK18, PARK21, PARK22, PARK23, PARK3, PARK7, PINK1, PLA2G6, PRKN, PRKRA, RAB39B, SCA2, SLC20A2, SLC30A10, SLC6A3, SNCA, SNCB, SPR, SYNJ1, TAF1, TH, TMEM230, TNK2, TNR, UCHL1, VPS13A, VPS35, WDR45, ARSD.
**Mitocarta genes** (1158 genes)		AADAT, AARS2, AASS, ABAT, ABCA13, ABCA9, ABCB10, ABCB6, ABCB7, ABCB8, ABCB9, ABCD1, ABCD2, ABCD3, ABCF2, ABHD10, ABHD11, ACAA1, ACAA2, ACACA, ACACB, ACAD10, ACAD11, ACAD8, ACAD9, ACADL, ACADM, ACADS, ACADSB, ACADVL, ACAT1, ACCS, ACLY, ACO1, ACO2, ACOT13, ACOT2, ACOT7, ACOT9, ACOX1, ACOX3, ACP6, ACSF2, ACSF3, ACSL1, ACSL4, ACSL6, ACSM1, ACSM2A, ACSM3, ACSM5, ACSS1, ACSS3, ACYP2, ADCK1, ADCK2, ADCK5, ADHFE1, AFG1L, AFG3L2, AGK, AGMAT, AGPAT5, AGR2, AGXT, AGXT2, AHCYL1, AIFM1, AIFM2, AIFM3, AK2, AK3, AK4, AKAP1, AKAP10, AKR1B10, AKR7A2, ALAS1, ALAS2, ALDH18A1, ALDH1B1, ALDH1L1, ALDH1L2, ALDH2, ALDH3A2, ALDH4A1, ALDH5A1, ALDH6A1, ALDH7A1, ALDH9A1, ALKBH1, ALKBH3, ALKBH7, AMACR, AMT, ANGEL2, APEX2, APOO, APOOL, APOPT1, ARF5, ARG2, ARL2, ARMC10, ARMS2, ASAH2, ATAD1, ATAD3A, ATAD3B, ATIC, ATP10D, ATP23, ATP5F1A, ATP5F1B, ATP5F1C, ATP5F1D, ATP5F1E, ATP5IF1, ATP5MC1, ATP5MC2, ATP5MC3, ATP5MD, ATP5ME, ATP5MF, ATP5MF-PTCD1, ATP5MG, ATP5MPL, ATP5PB, ATP5PD, ATP5PF, ATP5PO, ATP6, ATP8, ATPAF1, ATPAF2, ATXN2, AUH, AURKAIP1, BAD, BAK1, BAX, BCAT2, BCKDHA, BCKDHB, BCKDK, BCL2, BCL2L1, BCL2L13, BCL2L2, BCS1L, BDH1, BID, BLOC1S1, BNIP3, BNIP3L, BOLA1, BOLA3, BPHL, C12orf10, C12orf65, C15orf40, C15orf48, C15orf61, C16orf91, C1QBP, C2orf69, C3orf33, C5orf63, C6orf136, C6orf203, C8orf82, CA5A, CA5B, CARS2, CASP8, CAT, CBR3, CBR4, CCDC127, CCDC51, CCDC58, CCDC90B, CCT7, CDC25C, CEP89, CHCHD1, CHCHD10, CHCHD2, CHCHD3, CHCHD4, CHCHD5, CHCHD6, CHCHD7, CHDH, CHMP1B, CHPT1, CISD1, CISD2, CISD3, CKMT1A, CKMT1B, CKMT2, CLIC4, CLPB, CLPP, CLPX, CLYBL, CMC1, CMC2, CMC4, CMPK2, COA1, COA3, COA4, COA5, COA6, COA7, COASY, COMT, COMTD1, COQ10A, COQ10B, COQ2, COQ3, COQ4, COQ5, COQ6, COQ7, COQ8A, COQ8B, COQ9, COX10, COX11, COX14, COX15, COX16, COX17, COX18, COX19, COX2, COX20, COX4I1, COX4I2, COX5A, COX5B, COX6A1, COX6A2, COX6B1, COX6B2, COX6C, COX7A1, COX7A2, COX7A2L, COX7B, COX7C, COX8A, COX8C, CPOX, CPS1, CPT1A, CPT1B, CPT1C, CPT2, CRAT, CRLS1, CROT, CRY1, CRYZ, CS, CYB5A, CYB5B, CYB5R2, CYB5R3, CYC1, CYCS, CYP11A1, CYP11B2, CYP24A1, CYP27A1, CYP27B1, CYTB, D2HGDH, DAP3, DARS2, DBI, DBT, DCAKD, DCXR, DDAH1, DDX28, DECR1, DELE1, DEPP1, DGLUCY, DGUOK, DHCR24, DHODH, DHRS1, DHRS4, DHRS7B, DHRSX, DHTKD1, DHX30, DIABLO, DLAT, DLD, DLST, DMAC2, DMAC2L, DMGDH, DMPK, DNA2, DNAJA3, DNAJC11, DNAJC15, DNAJC19, DNAJC28, DNAJC30, DNAJC4, DNLZ, DNM1L, DTYMK, DUS2, DUSP26, DUT, EARS2, ECH1, ECHDC1, ECHDC2, ECHDC3, ECHS1, ECI1, ECI2, ECSIT, EEFSEC, EFHD1, EHHADH, ELAC2, EMC2, ENDOG, EPHX2, ERAL1, ETFA, ETFB, ETFDH, ETFRF1, ETHE1, EXOG, FABP1, FAHD1, FAHD2A, FAM136A, FAM162A, FAM185A, FAM210A, FAM210B, FARS2, FASN, FASTK, FASTKD1, FASTKD2, FASTKD3, FASTKD5, FBXL4, FDPS, FDX1, FDX2, FDXR, FECH, FH, FHIT, FIS1, FKBP10, FKBP8, FLAD1, FMC1-LUC7L2, FOXRED1, FPGS, FTH1, FTMT, FUNDC1, FUNDC2, FXN, GADD45GIP1, GAPDH, GARS, GATB, GATC, GATD3A, GATM, GCAT, GCDH, GCSH, GDAP1, GFER, GFM1, GFM2, GHITM, GK, GLDC, GLOD4, GLRX2, GLRX5, GLS, GLS2, GLUD1, GLYAT, GLYCTK, GNG5, GOLPH3, GOT2, GPAM, GPD2, GPI, GPT2, GPX1, GPX4, GRHPR, GRPEL1, GRPEL2, GRSF1, GSR, GSTK1, GSTO1, GSTZ1, GTPBP10, GTPBP3, GTPBP6, GUF1, GUK1, HADH, HADHA, HADHB, HAGH, HAO2, HARS2, HCCS, HDHD3, HDHD5, HEBP1, HEMK1, HIBADH, HIBCH, HIGD1A, HIGD2A, HINT1, HINT2, HINT3, HK1,

		HK2, HMBS, HMGCL, HMGCS2, HOGA1, HSCB, HSD17B10, HSD17B4, HSD17B8, HSDL1, HSDL2, HSPA9, HSPB7, HSPD1, HSPE1, HTATIP2, HTRA2, IARS2, IBA57, IDE, IDH1, IDH2, IDH3A, IDH3B, IDH3G, IDI1, IFI27, IMMP1L, IMMP2L, IMMT, ISCA1, ISCA2, ISCU, ISOC2, IVD, KARS, KIAA0100, KIAA0391, KIF1B, KMO, KRT5, KYAT3, L2HGDH, LACTB, LACTB2, LAMC1, LAP3, LARS2, LDHAL6B, LDHB, LDHD, LETM1, LETM2, LETMD1, LIAS, LIPT1, LIPT2, LONP1, LONP2, LRPPRC, LYPLA1, LYPLAL1, LYRM1, LYRM2, LYRM4, LYRM7, LYRM9, MACROD1, MAIP1, MALSU1, MAOA, MAOB, MARC1, MARC2, MARCH5, MARS2, MAVS, MCAT, MCCC1, MCCC2, MCEE, MCRIP2, MCU, MCUB, MCUR1, MDH1, MDH2, ME1, ME2, ME3, MECR, METAP1D, METTL15, METTL17, METTL5, METTL8, MFF, MFN1, MFN2, MGARP, MGME1, MGST1, MGST3, MICOS10, MICOS13, MICU1, MICU2, MIEF1, MIPEP, MLH1, MLYCD, MMAB, MMACHC, MMADHC, MMUT, MOCS1, MPC1, MPC1L, MPC2, MPST, MPV17, MPV17L, MPV17L2, MRM1, MRM2, MRM3, MRPL1, MRPL10, MRPL11, MRPL12, MRPL13, MRPL14, MRPL15, MRPL16, MRPL17, MRPL18, MRPL19, MRPL2, MRPL20, MRPL21, MRPL22, MRPL23, MRPL24, MRPL27, MRPL28, MRPL3, MRPL30, MRPL32, MRPL33, MRPL34, MRPL35, MRPL36, MRPL37, MRPL38, MRPL39, MRPL4, MRPL40, MRPL41, MRPL42, MRPL43, MRPL44, MRPL45, MRPL46, MRPL47, MRPL48, MRPL49, MRPL50, MRPL51, MRPL52, MRPL53, MRPL54, MRPL55, MRPL57, MRPL58, MRPL9, MRPS10, MRPS11, MRPS12, MRPS14, MRPS15, MRPS16, MRPS17, MRPS18A, MRPS18B, MRPS18C, MRPS2, MRPS21, MRPS22, MRPS23, MRPS24, MRPS25, MRPS26, MRPS27, MRPS28, MRPS30, MRPS31, MRPS33, MRPS34, MRPS35, MRPS36, MRPS5, MRPS6, MRPS7, MRPS9, MRRF, MRS2, MSRA, MSRB2, MSRB3, MTCH1, MTCH2, MTCP1, MTERF1, MTERF2, MTERF3, MTERF4, MTFMT, MTFP1, MTFR1, MTFR1L, MTG1, MTG2, MTHFD1, MTHFD1L, MTHFD2, MTHFD2L, MTHFS, MTIF2, MTIF3, MTO1, MTPAP, MTRF1, MTRF1L, MTX1, MTX2, MUL1, MUTYH, NADK2, NAGS, NARS2, NAXD, NAXE, NBR1, NCEH1, NCOA4, ND1, ND2, ND3, ND4, ND4L, ND5, ND6, NDUFA1, NDUFA10, NDUFA11, NDUFA12, NDUFA13, NDUFA2, NDUFA3, NDUFA4, NDUFA5, NDUFA6, NDUFA7, NDUFA8, NDUFA9, NDUFAB1, NDUFAF1, NDUFAF2, NDUFAF3, NDUFAF4, NDUFAF5, NDUFAF6, NDUFAF7, NDUFAF8, NDUFB1, NDUFB10, NDUFB11, NDUFB2, NDUFB3, NDUFB4, NDUFB5, NDUFB6, NDUFB7, NDUFB8, NDUFB9, NDUFC1, NDUFC2, NDUFS1, NDUFS2, NDUFS3, NDUFS4, NDUFS5, NDUFS6, NDUFS7, NDUFS8, NDUFV1, NDUFV2, NDUFV3, NEU4, NFS1, NFU1, NGRN, NIF3L1, NIPSNAP1, NIPSNAP2, NIPSNAP3A, NIPSNAP3B, NIT1, NIT2, NLN, NLRX1, NME3, NME4, NME6, NMNAT3, NNT, NOA1, NPL, NRDC, NSUN3, NSUN4, NT5C, NT5DC2, NT5DC3, NT5M, NTHL1, NUBPL, NUCB2, NUDT13, NUDT19, NUDT2, NUDT5, NUDT6, NUDT8, NUDT9, OAT, OBSCN, OCIAD1, OCIAD2, OGDH, OGDHL, OGG1, OMA1, OPA1, OPA3, OSBPL1A, OSGEPL1, OTC, OXA1L, OXCT1, OXLD1, OXNAD1, OXR1, OXSM, P4HB, PABPC5, PACSIN2, PAICS, PAK5, PAM16, PANK2, PARK7, PARL, PARS2, PC, PCBD2, PCCA, PCCB, PCK2, PDE12, PDF, PDHA1, PDHA2, PDHB, PDHX, PDK1, PDK2, PDK3, PDK4, PDP1, PDP2, PDPR, PDSS1, PDSS2, PET100, PET117, PEX11B, PGAM5, PGS1, PHB, PHB2, PHYH, PI4KA, PICK1, PIF1, PINK1, PISD, PITRM1, PKLR, PLGRKT, PLPBP, PMAIP1, PMPCA, PMPCB, PNPLA8, PNPO, PNPT1, POLDIP2, POLG, POLG2, POLRMT, PPA2, PPIF, PPM1K, PPOX, PPTC7, PPWD1, PRDX2, PRDX3, PRDX4, PRDX5, PRDX6, PRELID1, PRELID2, PRELID3A, PRELID3B, PREPL, PRODH, PRODH2, PRSS35, PRXL2A, PSMA6, PSTK, PTCD1, PTCD2, PTCD3, PTGES2, PTGS1, PTPMT1, PTPN4, PTRH1, PTRH2, PTS, PUS1, PUSL1, PXMP2, PXMP4, PYCR1, PYCR2, PYURF, QDPR, QRSL1, QTRT1, RAB11FIP5, RAB24, RAB32, RAB35, RAB5IF, RARS, RARS2, RBFA, RCC1L, RCN2, RDH11, RDH13, RDH14, RECQL4, REXO2, RFK, RHOT1, RHOT2, RIDA, RMDN1, RMDN3, RMND1, RNASEH1, ROMO1, RPIA, RPL10A, RPL34, RPL35A, RPS14, RPS15A, RPS18, RPUSD3, RPUSD4, RRM2B, RSAD1, RTN4IP1, SAMM50, SARDH, SARS2, SCCPDH, SCO1, SCO2, SCP2, SDHA, SDHAF1, SDHAF2, SDHAF3, SDHAF4, SDHB, SDHC, SDHD, SDR39U1, SDSL, SECISBP2, SELENOO, SERAC1, SERHL2, SETD9, SFXN1, SFXN2, SFXN3, SFXN4, SFXN5, SHMT1, SHMT2, SIRT3, SIRT4, SIRT5, SLC16A1, SLC16A11, SLC16A7, SLC22A4, SLC25A1, SLC25A10, SLC25A11, SLC25A12, SLC25A13, SLC25A14, SLC25A15, SLC25A16, SLC25A17, SLC25A18, SLC25A19, SLC25A20, SLC25A21, SLC25A22, SLC25A23, SLC25A24, SLC25A25, SLC25A26, SLC25A27, SLC25A28, SLC25A29, SLC25A3, SLC25A30, SLC25A31, SLC25A32, SLC25A33,

		SLC25A34, SLC25A35, SLC25A36, SLC25A37, SLC25A38, SLC25A39, SLC25A4, SLC25A40, SLC25A41, SLC25A42, SLC25A43, SLC25A44, SLC25A45, SLC25A46, SLC25A47, SLC25A48, SLC25A5, SLC25A51, SLC25A53, SLC25A6, SLC30A6, SLC30A9, SLC37A4, SLIRP, SMDT1, SNAP29, SND1, SOD1, SOD2, SPATA19, SPATA20, SPG7, SPR, SPRYD4, SPTLC2, SQOR, SSBP1, STAR, STARD7, STOM, STOML1, STOML2, STX17, SUCLA2, SUCLG1, SUCLG2, SUGCT, SUOX, SUPV3L1, SURF1, SYNJ2BP, TACO1, TAMM41, TARS, TARS2, TBRG4, TCAIM, TCHP, TCIRG1, TDRKH, TEFM, TFAM, TFB1M, TFB2M, THEM4, THG1L, THNSL1, TIMM10, TIMM10B, TIMM13, TIMM17A, TIMM17B, TIMM21, TIMM22, TIMM23, TIMM29, TIMM44, TIMM50, TIMM8A, TIMM8B, TIMM9, TIMMDC1, TK2, TKT, TMBIM4, TMEM11, TMEM126A, TMEM126B, TMEM143, TMEM14C, TMEM177, TMEM186, TMEM205, TMEM65, TMEM70, TMLHE, TOMM20, TOMM22, TOMM34, TOMM40, TOMM40L, TOMM5, TOMM6, TOMM7, TOMM70, TOP1MT, TOP3A, TPI1, TRAP1, TRIAP1, TRIT1, TRMT1, TRMT10C, TRMT11, TRMT2B, TRMT61B, TRMU, TRNT1, TRUB2, TSFM, TSPO, TST, TSTD1, TSTD3, TTC19, TUBB3, TUFM, TXN2, TXNDC12, TXNRD1, TXNRD2, TYSND1, UCP1, UCP2, UCP3, UNG, UQCC1, UQCC2, UQCR10, UQCR11, UQCRB, UQCRC1, UQCRC2, UQCRFS1, UQCRH, UQCRQ, VARS2, VDAC1, VDAC2, VDAC3, VWA8, WARS2, WDR81, XPNPEP3, YARS2, YBEY, YME1L1, ZADH2.
