# Supplementary figures and images for: Over-Mutated Mitochondrial, Lysosomal and TFEB-Regulated Genes in Parkinson’s Disease
Source: J Clin Med. 2022 Mar 21;11(6):1749. doi: 10.3390/jcm11061749 (PMC8951534; doi:10.3390/jcm11061749)

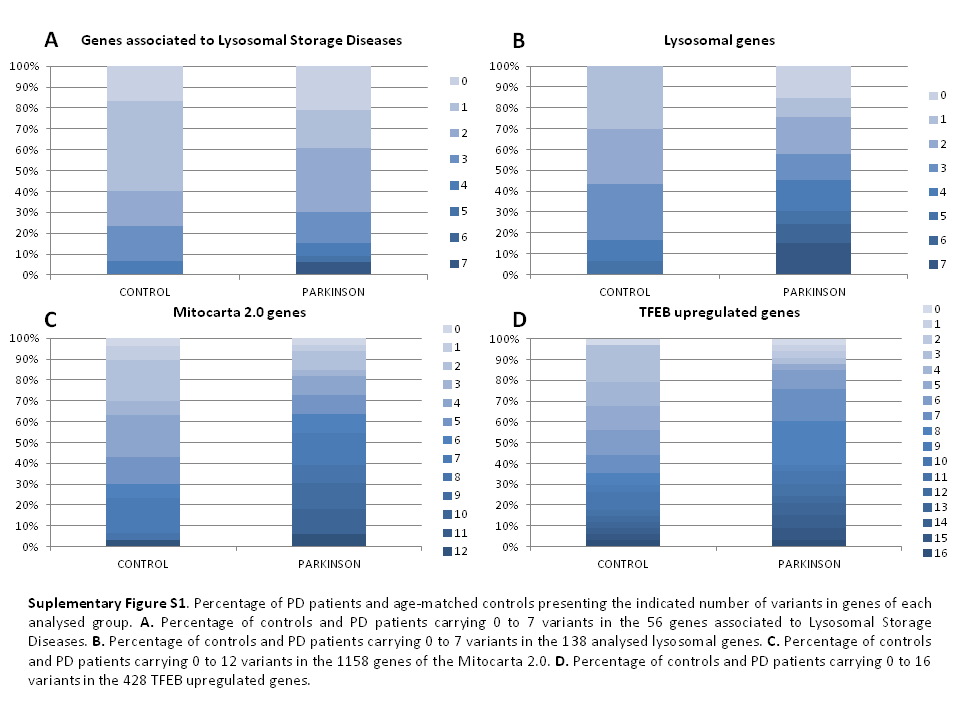

Supplement: Supplementary file 1 [file jcm-11-01749-s001.zip › Supl_Fig S1.tif]

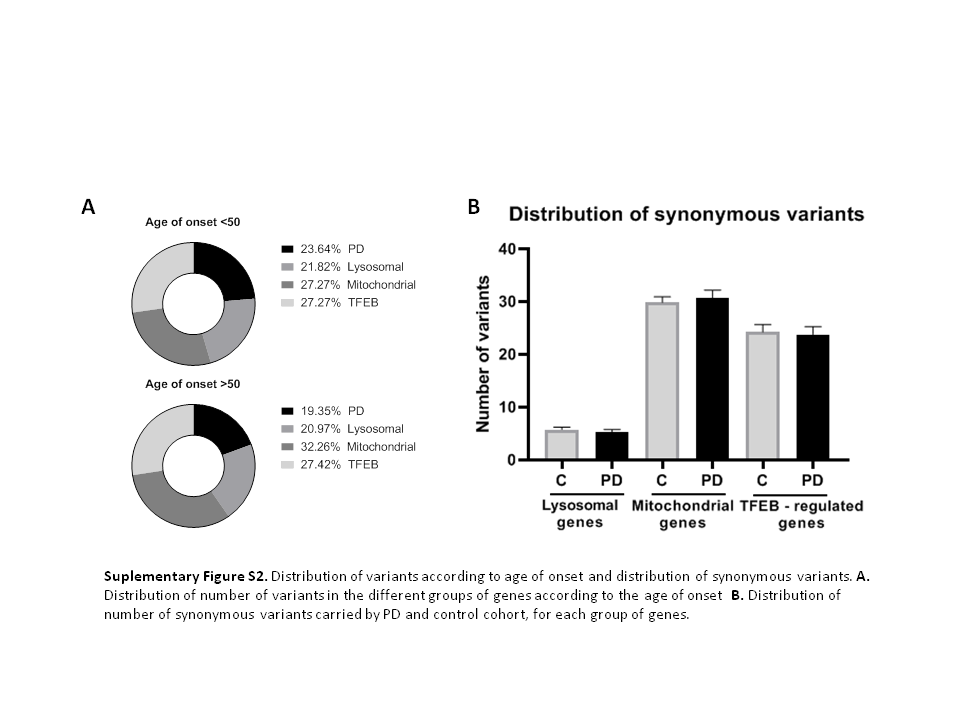

Supplement: Supplementary file 1 [file jcm-11-01749-s001.zip › Supl_Fig S2.tif]
